# Post COVID 19 Pandemic –Changing landscape of Dementia Profile A Study from a tertiary care center in India

**DOI:** 10.1002/alz.090865

**Published:** 2025-01-03

**Authors:** Suman Kushwaha, Ashwin Kumar Panda, Mridula Singh, Aldrin Anthony, Siddharth Maheshwari, Rashi Kumar, Rajinder Dhamija

**Affiliations:** ^1^ Institute of Human Behaviour and Allied Sciences, Delhi India; ^2^ IHBAS, New Delhi, New Delhi India; ^3^ IHBAS, New Delhi, New Delhi, Delhi India; ^4^ Institute of Human Behaviour and Allied Sciences, Delhi, Delhi India

## Abstract

**Background:**

SARS CoV‐2, neurotrophic virus in response to neuroinflammation causing varying cognitive deficits in post pandemic period. New onset rapidly progressive cognitive decline and deterioration in degenerative dementia has been seen in clinics in recent times. The paradigm shift of presentation of dementia needs detailed evaluation to facilitate further management.

**Method:**

Duration of study‐ 2 years. Retrospective

All the patients admitted with cognitive impairment were evaluated: Detailed history, Cognitive testing, biochemical and hematological parameters. Neuroimaging in all patients & CSF in selected cases. Accordingly categorized into subtypes. History of COVID 19 was confirmed by Clinically, hospitalization and investigations.

**Result:**

Total Cases – 53. Age Range 50 ‐75 yr. Dementia Subtypes Patients, Alzheimer’s disease –14, Frontotemporal ‐10, Rapidly progressive dementia ‐15, Vascular – 8 and Parkinson’s dementia‐ 6.

History of respiratory symptoms were present in 28 patients while COVID test was positive in 12 patients. Hospitalization for COVID ‐19 in 10 patients. The degenerative dementias have rapid progression of symptoms. The incidence of RPD has increased. Treatable cause of RPD were investgated 12 patient had immune mediated dementia and has partial response on immunomodulation. Seven patients have succumbed.

**Conclusion:**

COVID‐19 has significant effect on cognitive decline. Neuroinflammation is key factor causing degeneration. We need to know how this is resulting in neurodegenerative processes and cognitive functioning. There is need to further explore post pandemic dementia in systematic studies for new onset cognitive decline for facilitating the therapeutic strategies.

